# Cortical Thickness Mapping to Identify Focal Osteoporosis in Patients with Hip Fracture

**DOI:** 10.1371/journal.pone.0038466

**Published:** 2012-06-11

**Authors:** Kenneth E. S. Poole, Graham M. Treece, Paul M. Mayhew, Jan Vaculík, Pavel Dungl, Martin Horák, Jan J. Štěpán, Andrew H. Gee

**Affiliations:** 1 Department of Medicine, University of Cambridge, Cambridge, Cambridgeshire, United Kingdom; 2 Department of Engineering, University of Cambridge, Cambridge, Cambridgeshire, United Kingdom; 3 Department of Orthopaedics, Faculty of Medicine, Charles University Prague and Bulovka Hospital, Prague, Czech Republic; 4 Department of Radiology, Homolka Hospital, Prague, Czech Republic; 5 Department of Rheumatology, Faculty of Medicine 1, Charles University Prague and Institute of Rheumatology, Prague, Czech Republic; Georgia Health Sciences University, United States of America

## Abstract

**Background:**

Individuals with osteoporosis are predisposed to hip fracture during trips, stumbles or falls, but half of all hip fractures occur in those without generalised osteoporosis. By analysing ordinary clinical CT scans using a novel cortical thickness mapping technique, we discovered patches of markedly thinner bone at fracture-prone regions in the femurs of women with acute hip fracture compared with controls.

**Methods:**

We analysed CT scans from 75 female volunteers with acute fracture and 75 age- and sex-matched controls. We classified the fracture location as femoral neck or trochanteric before creating bone thickness maps of the outer ‘cortical’ shell of the intact contra-lateral hip. After registration of each bone to an average femur shape and statistical parametric mapping, we were able to visualise and quantify statistically significant foci of thinner cortical bone associated with each fracture type, assuming good symmetry of bone structure between the intact and fractured hip. The technique allowed us to pinpoint systematic differences and display the results on a 3D average femur shape model.

**Findings:**

The cortex was generally thinner in femoral neck fracture cases than controls. More striking were several discrete patches of statistically significant thinner bone of up to 30%, which coincided with common sites of fracture initiation (femoral neck or trochanteric).

**Interpretation:**

Femoral neck fracture patients had a thumbnail-sized patch of focal osteoporosis at the upper head-neck junction. This region coincided with a weak part of the femur, prone to both spontaneous ‘tensile’ fractures of the femoral neck, and as a site of crack initiation when falling sideways. Current hip fracture prevention strategies are based on case finding: they involve clinical risk factor estimation to determine the need for single-plane bone density measurement within a standard region of interest (ROI) of the femoral neck. The precise sites of focal osteoporosis that we have identified are overlooked by current 2D bone densitometry methods.

## Introduction

**Figure 1:**
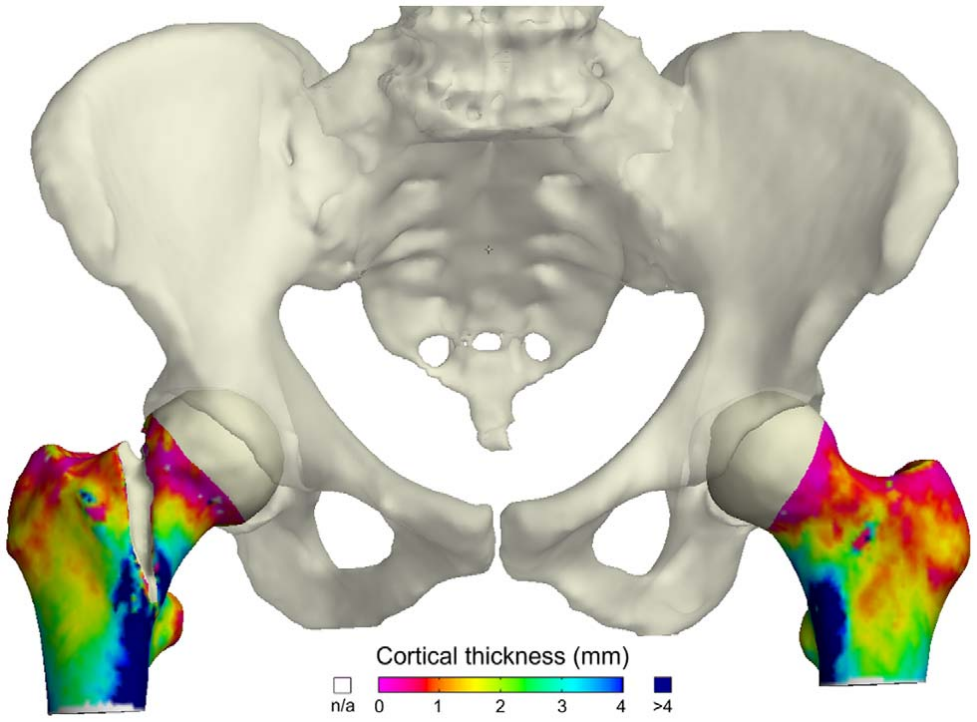
Cortical Thickness Colour Mapping using ordinary clinical CT data. Femora and pelvis from an 84-year-old osteoporotic female who sustained a fracture without falling. She felt her right hip break as she placed her right foot on a low step. Femoral neck BMD was 0.46 g/cm2, T score −3.3. From the Arthritis Research UK FEMCO study (07/H0305/61).

The annual incidence of hip fractures is projected to rise fourfold to 6.3 million worldwide by 2050, because of the exponentially increasing risk of fracture as people live longer. Studying femoral neck and trochanteric fractures is therefore a health priority [1]. In older people, the proximal femur breaks when the loads placed on it overcome its strength, with common loading scenarios being sideways falls, stumbles or sudden unusual movements [2]. However, a spontaneous or ‘impact-free’ mechanism accounts for up to 6% of hip fractures (fig. 1) [3], [4]. We know that women with osteoporosis (who have generally thinner and more porous bones) are more likely to suffer hip fracture, but most people who will sustain hip fracture do not have generalised osteoporosis [5]. We also know that the outer ‘cortical’ bone of the femur where fractures initiate [6] thins rapidly with age [7], [8], is a key determinant of bone strength and fracture risk [9]–[13] and responds well to certain osteoporosis drugs [14], [15]. Here we ask; Is there a pattern of femoral bone thinning common to hip fracture patients and, if so, is it generalised or focal? Could focal osteoporosis of the femur be a cause of hip fracture in the elderly? The answer to these questions might illuminate why hip fractures tend to initiate in particular zones (fig. 1).

A new CT image processing technique [16] allows us to display cortical thickness as a colour map over the bone surface, with several thousand independent measurements across each proximal femur and sufficient sensitivity to detect even small differences (∼30 microns) when expressed systematically by a suitably sized cohort. We use it here to pinpoint differences in bone thickness between women with and without hip fracture. We examined the contra-lateral side as a surrogate for the broken hip in these female fracture patients, having previously identified symmetry in femoral neck cortical thickness [8].

## Methods

From 2006 to 2009, women admitted to Bulovka University Hospital, Prague with an acute hip fracture were consented to the pragmatic ‘Surgical treatment of the hip joint in trauma’ study (PI Professor P Dungl), part of which involved a clinical CT scan of both hips before surgical fixation [17]. Participants were positioned on the Siemens two-compartment Osteo phantom and a single CT scan (either Siemens Sensation 40 or 16 detector, B10/20 kernel, ≤1 mm reconstructed slice thickness) was performed including both hips from above the acetabulum to just below the lesser trochanter. Women were aged over 50, were awaiting surgical repair of a cervical or trochanteric fracture and had sustained a low energy injury. Women were excluded if they had metalwork in either hip, high trauma injury, metastatic cancer, unilateral bone disease, subtrochanteric fracture or terminal illness. Using the same criteria, a convenience control sample of older women without fracture was recruited by invitation at rheumatology clinics and two residential care centres in the same districts of Prague. From 204 invitations, 108 fracture-free women responded of whom 81 were eligible for CT scanning at Homolka hospital, Prague (Siemens Sensation 16 detector B20 kernel, ≤1mm reconstructed slice thickness). At the image quality control step, 6 scans were excluded (due to insufficient scan length or undisclosed metalwork) leaving 75 female controls. One age-matched case was selected for each of the 75 eligible control participants, from the total sample of 242 women with hip fracture. Where precise birth year age matching was not possible, the next nearest matching case was selected up to a maximum 5-year age difference. The final sample taken forward for cortical thickness mapping comprised 150 femurs from 75 women in each group (mean ages of femoral neck fracture cases 78.1+/−7.1 years, trochanteric fracture cases 75.2+/−7.9, controls 76.6+/−7.3 years). There were 36 femoral neck fractures and 39 trochanteric fractures.

**Figure 2:**
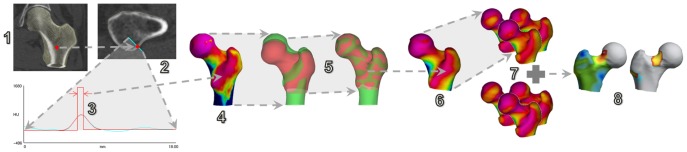
Cortical thickness analysis. 1. Measurements are performed at every vertex in an approximate segmentation of the hip. 2. At each vertex, the CT data is sampled on a line passing through the cortex. 3. A model-based fit is used to estimate the cortical thickness, allowing for image blur. 4. The thickness is mapped back to the surface (here blue is thick, pink is thin). 5. An average femur (red) is deformed to match the current femur (green). 6. Thickness estimates are then transferred to the average femoral surface and smoothed. 7. This process is repeated for all subjects, producing subject-specific thickness estimates all mapped to the same, average surface. 8. The data is analysed using statistical parametric mapping, to obtain mean thickness differences between groups and also the significance of these differences.

**Figure 3:**
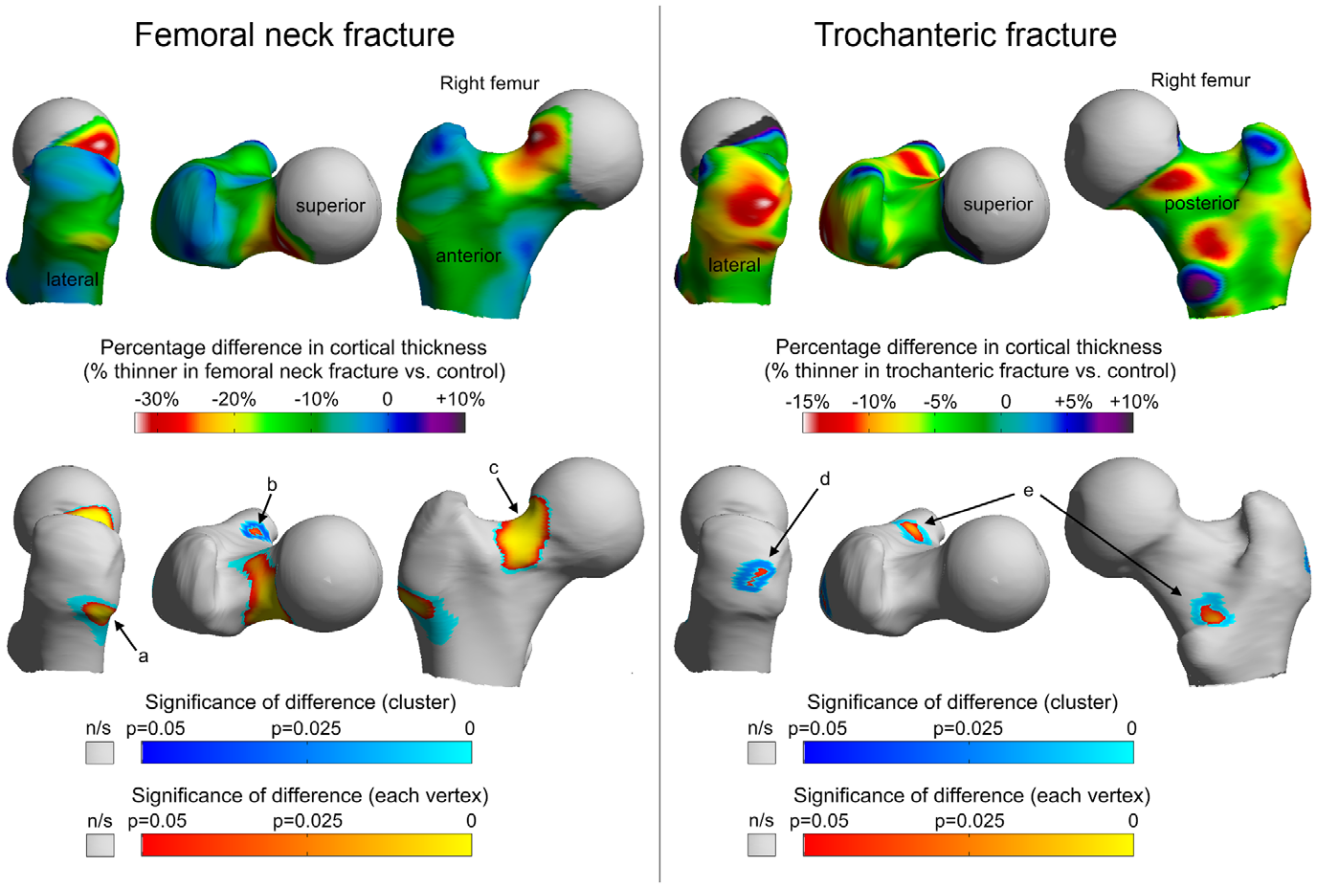
Results for femoral neck fracture (left) and trochanteric fracture (right). Upper colour maps show the average percentage difference in cortical thickness for each fracture type versus control (displayed on an average right femur model). The lower colour maps are the significance of the differences adjusted for age, height and weight, either point by point (vertex) or as a whole patch (blue clusters). Note that all the blue clusters extend uninterrupted beneath their respective orange/yellow vertices. Table 1 gives adjusted thickness values and significance of the clusters a–e.

The analysis method is illustrated in figure 2. Anonymised axial dicom images were received in Cambridge via the secure DICOM internet connection ePACS (ICZ, Brno, Czech Rep.) where they were reconstructed to classify fracture side and site according to AO criteria. Standard clinical hip bone density (2D areal DXA-equivalent) was measured in the ‘total hip’ region of interest (ROI) of each femur using QCTpro software (v4.2.3 Mindways, Austin, Texas, USA). The unfractured contralateral hip (or matching side in controls) was segmented semi-automatically in Stradwin v4.2 software (Treece, Gee, Cambridge) before mapping cortical thickness at approximately 6000 surface points per femur. Cortical thickness was estimated from the CT data using the method described by Treece et al. [16]. By making reasonable assumptions about both the anatomy and the imaging blur, thickness can be measured to super-resolution accuracy across the entire proximal femur, apart from at the femoral head where the proximity of the acetabulum is problematic. The methodology has been validated against thickness measurements obtained from high resolution micro-CT scans of cadaveric femurs [16]. Analysis of the 150 thickness maps followed established practice within the neuroimaging community, who have pioneered techniques for statistical inference from dense, spatially correlated data. To account for variations in inter-subject morphology, each map was spatially realigned with a canonical femur surface using a B-spline free-form deformation calculated by the iterative closest point registration algorithm [18]. The spatially normalized maps were then smoothed with a 10 mm full-width-half-maximum filter. We investigated differences in cortical thickness between i) femoral neck fractures (n36) and all controls or ii) trochanteric fractures (n39) and all controls. Formal inference was accomplished by statistical parametric mapping (SPM) [19], as implemented in the SurfStat package [20]. Model effects were group (case*control), age, height and weight. Missing height and weight values (2 fracture cases) were replaced with group mean values. T-statistics were calculated to test the significance of the group term. Random field theory then furnished p-values, corrected for multiple comparisons to control the overall image-wise chance of false positives. Figure 3 shows corrected p maps based on the magnitude of peaks (sensitive to focal effects) and on the extent of connected clusters exceeding an uncorrected p-value threshold of 0.001 (sensitive to distributed effects). All participants with fracture gave written informed consent. Control participants gave verbal consent which was documented in the medical notes as agreed with the Ethics Committee. Ethics committees approved the study in the Czech Republic (Ethical Committee of the Institute of Rheumatology and Ethical Committee of Bulovka Hospital, ref IRB0002384101) and in the UK (Cambridgeshire 4, ref 07/H0305/61).

**Table 1:** Details of thinner patches of femoral cortex in hip fracture.

*Hip fracture type*	Location of ‘cluster’ Patch where bone cortex was thinner in hip fracture cases	Mean adjusted cortical thickness in cluster (Cases)		Mean adjusted cortical thickness in cluster (Cluster)		p value for difference
		Millimetres	±SD	Millimetres	±SD	
***Femoral neck fractures***						
Patch a (fig.3a)	Greater trochanter	1.14	0.15	1.34	0.26	0.00407
Patch b (fig.3b)	Lesser trochanter	0.85	0.16	0.98	0.19	0.0319
Patch c (fig.3c)	Head-neck junction	0.62	0.10	0.77	0.14	0.00000350
***Trochanteric fractures***						
Patch d (fig.3d)	Greater trochanter	1.05	0.25	1.21	0.27	0.0237
Patch e (fig.3e)	Lesser trochanter	0.78	0.15	0.88	0.17	0.0108

## Results

Percentage differences in cortical thickness between each hip fracture group and the control group were displayed on an average right femur surface map using a colour scale. Views from several anatomical planes were chosen to illustrate the differences (femoral neck fractures vs. controls; fig. 3 *left upper panel* and trochanteric fractures vs controls; fig. 3 *right upper panel*). Similar maps were created to visualise the statistical significance of differences (fig 3. *lower panels)*. Several distinct patches of up to 30% thinner cortical bone were identified in fracture cases which coincided with typical sites of hip fracture. No regions of statistically significant thicker bone were seen in fracture cases. WHO-defined osteoporosis (a total hip DXA-equivalent bone mineral density T score <−2.5) was present in less than half of hip fracture patients (31/75, 41.3%) and 9/75 (12%) controls. The age, height and weight adjusted values for the clusters of thinner bone associated with each fracture type are shown in table 1. The mean, unadjusted value of whole proximal femur cortical thickness among femoral neck fracture patients was 1.20 mm±0.17 mm, compared with a value of 1.25 mm±0.20 mm among trochanteric fracture patients and 1.30 mm±0.21 mm among controls (ANOVA p = 0.0388). Whole femur cortical thickness was statistically significantly lower in femoral neck fracture compared with control (Dunnett’s values* were 0.012 for neck fracture, p = 0.024, and −0.04 for trochanteric fracture, p = 0.34; *[absolute difference in sample means] - [least significant difference]). The age and weight terms were significant within the 150 femurs (age range 55–98 and weight range 40–89 kg). Significant thinning of approximately 0.02 mm per year from age 55–98 was apparent in the infero-medial region. Significant thickening of approximately 0.02 mm per kilogram was evident in a similar infero-medial region.

## Discussion

**Figure 4:**
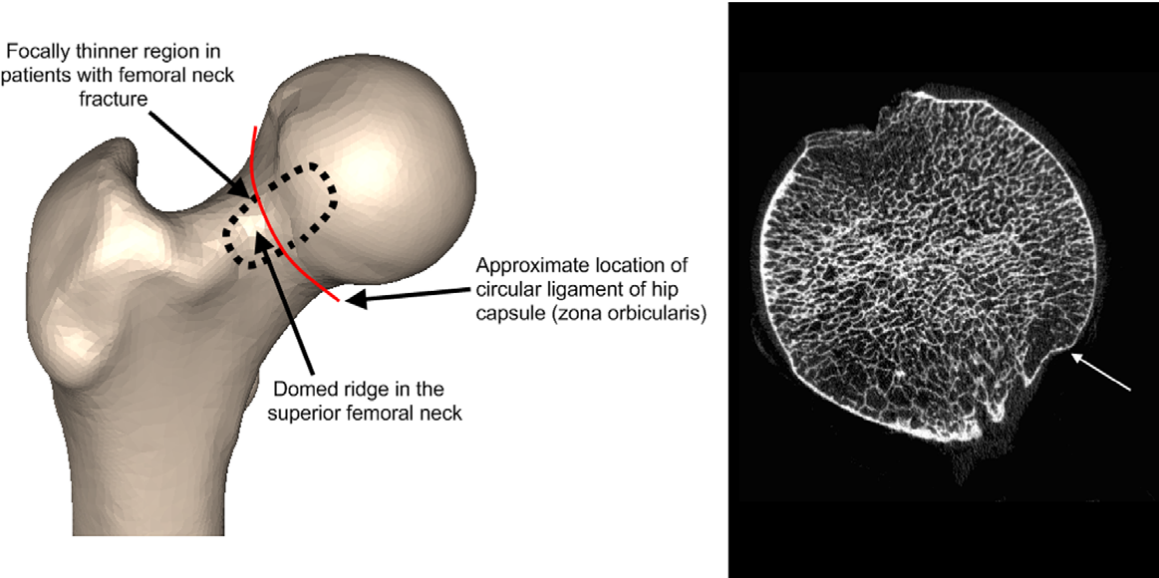
Anatomical context of focal thinning in women with femoral neck fracture. The left pane is a right proximal femur model seen from the front. The thin patch of cortex (fig 3c) in femoral neck fracture patients occurs on the domed ridge called the femoral neck eminence [25]. The right pane is a high resolution CT image through the femoral head of a 90 year old female (aBMD total hip T-score −1.9) which suggests that the patch is osteoporotic with microarchitectural thinning (white arrow). Femur courtesy of the Melbourne Femur Collection, Chairman Professor John Clement (Melbourne Dental School).

**Figure 5:**
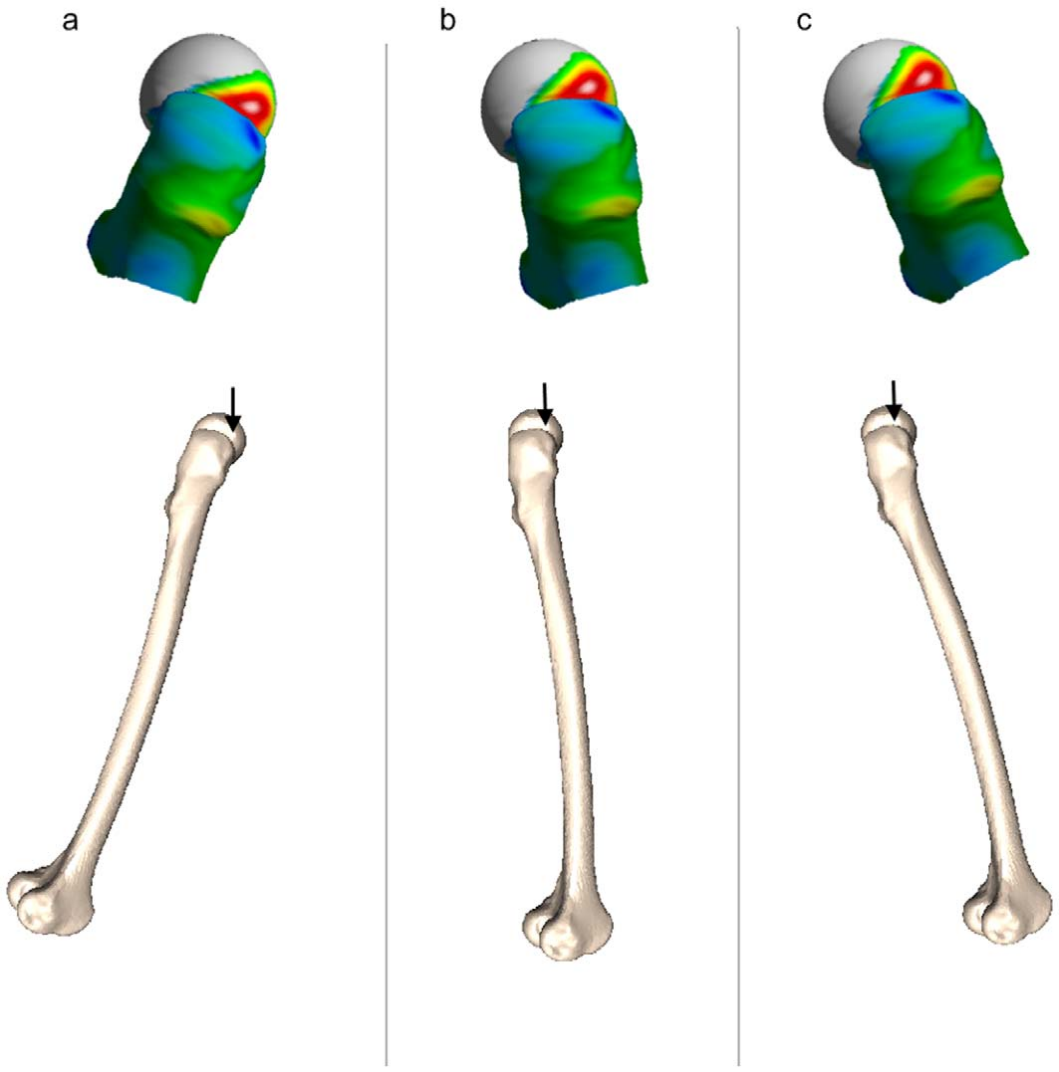
Approximate orientation of the focally thin (red) zone during the different phases of gait. Right femur (a) toe-off, (b) single leg stance and (c) heel strike. A slightly modified stance position (b) conferred the greatest risk of spontaneous femoral neck fracture in the laboratory simulations of Cristofolini et al [3]. The focally thin patch we identified coincides with a region of high tensile stress during simulations of spontaneous fracture.

We used cortical thickness mapping to explore differences between women with and without recent hip fracture and identified generalised thinning of the femoral cortex in fracture patients. We also discovered focal differences manifest as several well-defined patches of markedly thinner femoral cortex in hip fracture patients compared to controls. Since osteoporosis is defined as microarchitectural deterioration of bone tissue, we consider that these areas of focally thinner bone are best described as patches of focal osteoporosis (fig. 4, right panel). The patches were evident at common sites involved in fracture, the most severe being a thumbnail-sized patch of up to 30% thinner bone at the head-neck junction in patients with femoral neck fracture (figs. 3, 4 and 5). Focal osteoporosis at the head-neck junction may play an important role in fractures associated with falls, and might even be involved in ‘spontaneous’ hip fracture on rare occasions. While the locations of the patches of focal osteoporosis appear to be critical in determining fracture type, we cannot judge whether they are involved in causing hip fracture, which requires prospective research. However it is noteworthy that among all the bone structural parameters measured in the largest prospective study of hip CT in older men and women conducted to date, cortical thickness estimates from a supero-anterior part of the femoral neck were the best predictor of subsequent hip fracture [9].

We assume that a fall onto or near the hip was the principal fracture mechanism in these women, but we did not routinely collect information on how these women fell, a priority for future work. The largest patch of thinner femoral cortex that we identified (fig 3c) appears to correspond to a key site of fracture initiation in a simulation of femoral neck fracture during a sideways fall to the ground [21]. Although the women we studied with trochanteric fracture also had patches of thinner bone in fracture-relevant zones (fig. 3d and 3e), it is not clear whether they correspond to trochanteric fracture initiation sites in the relevant simulations [21]. Nevertheless, it is interesting to note that the focally thin bone in the lateral facet of the greater trochanter (fig. 3d) in the trochanteric fracture patients coincided with one of the insertion sites of gluteus medius, which receives considerable force during locomotion.

While most hip fractures in the elderly are a result of injurious falls, spontaneous fractures of the femoral neck prior to falls have been implicated in up to 6% of cases, translating to more than 4000 hip fractures annually in the UK [22]. Previously, several research groups have reproduced these impact-free hip fractures in cadaveric femurs by simulating either the effects of increasing loads in stance or sudden large hip flexor muscle contractions. In Cristofolini’s specimens, increasing the load by simulating one-legged stance (as in fig. 5b), led to crack initiation at the junction between the femoral head and neck, with the subsequent catastrophic failure of the femur closely resembling that observed in our patient with impact-free fracture (fig. 1) [3]. Likewise simulating sudden psoas muscle contraction (as happens when a person attempts to stabilise their trunk on a fixed leg during a slip or stumble) led to subcapital fracture in a similar location [23]. The conserved patch of focal osteoporosis we identified among our femoral neck fracture patients (fig. 3c) appears to correspond with the sites of high tensile stress induced in those simulations. In the light of our findings we wonder what effect osteoporosis medicines might have on the thin patches of bone and in particular if strengthening the thin areas could prevent stumbling-induced or spontaneous hip fractures. Analysis of large clinical trials with serial CT is needed to address this question.

We are currently unable to answer a key question generated by these results; namely how did the focal patches of thin cortex arise? Several intriguing ideas come from histological and macroscopic studies of the head-neck junction in patients with fracture and from cadavers. Freeman et al. discovered that in fracture specimens, the underlying bone from the head-neck junction frequently contains microcallus, considered to be evidence of tensile fatigue damage [24]. Modelling the behaviour of the head-neck junction during habitual locomotion and falls is therefore a priority for biomechanics research. Although there were marked age and weight effects within the women we studied, the decreasing cortical thickness associated with age and increasing thickness associated with weight affected the inferior femur; i.e. on the opposite side of the femoral neck to the patch of focally thin bone. Thus we assume that neither younger nor heavier women were necessarily protected from having a thin cortex at the head-neck junction. The patch of focal osteoporosis in femoral neck fracture patients corresponds macroscopically with the junction between femoral head cartilage and bone, and tracks along the domed ridge running along the top of the femoral neck (called the femoral neck eminentia, or eminence [25], fig. 4). Since the thin cortical bone is so well circumscribed at this site, we concur with Panzer et al. in describing the differences as ‘focal osteoporosis’, but acknowledge that higher resolution and histological studies would be useful to further characterise the cortical and sub-cortical bone [26]. The circular fibres of the hip capsule (the zona orbicularis) also encircle the femoral neck at the focally thin patch. Pitt and others described a mechanical, abrasive action of the overlying hip capsule, ligaments and psoas muscle at this patch that commonly results in a ‘reaction area’ with occasional underlying radiolucency. This lucency can be appreciated on plain x-rays and is known to radiologists as ‘Pitt’s Pit’ [27]. Studies of the underlying histology of this zone in femoral neck fracture cases are clearly warranted [28], [29], and Pitt suggested that the zone could be involved in hip fracture pathogenesis.

This work has several weaknesses, namely pragmatic case selection, the use of a convenience sample of controls and reliance on the intact hip as a surrogate for the fractured hip. The results need replication in a better-characterised population sample, with particular attention to recalled injury mechanism. Statistical Parametric Mapping does not indicate causality; for instance it is possible (but unlikely) that controls could have substantial thickening of bone at various sites through unknown mechanisms. Finally, although studying the cortex is important in determining bone strength, alternative methods such as finite element (FE) models use whole bone biomechanics, and can therefore be informative in determining how and why individuals fracture their hips (as reviewed recently by Cristofolini et al [30]). In this regard, it is interesting to note that our cortical thickness maps have the potential to be converted into inner and outer surfaces for optimal delineation of cortical and trabecular compartments, which may help to improve the FE methods that currently assume a constant cortical thickness throughout the bone.

In related work, Li et al applied SPM to 3D density maps of femurs and discovered focal regions where clustered voxels of bone density differed significantly between hip fracture cases and controls [31]. In their analysis, several fracture-relevant density ROI’s showed promise in defining a hip fracture phenotype. The fact that Li’s head-neck junction femoral ROI based on volumetric density appears to coincide with the focally thinner femoral cortex we find at the head-neck junction suggests that having poor quality bone here is particularly concerning for future fracture risk. Previous prospective studies indicated that combining measures (e.g one measure of density, one of cortical thickness and one of bone shape) resulted in the optimum prediction of incident hip fracture [11], [32]. However, large prospective studies are necessary to determine what thresholds of cortical thickness or density in these newly discovered zones are predictive of hip fracture and might be a trigger for intervention in an individual. Our work is useful in defining ROI’s for cortical bone analysis that can then be taken forward for testing in prospective studies. Current hip fracture prevention strategies are based on case finding: they involve clinical risk factor estimation to determine the need for single plane bone density measurement within a standard femoral neck ROI. The precise sites of focal osteoporosis that we have now identified are overlooked by current 2D bone densitometry methods.
